# Aptamer-PEG-modified Fe_3_O_4_@Mn as a novel T1- and T2- dual-model MRI contrast agent targeting hypoxia-induced cancer stem cells

**DOI:** 10.1038/srep39245

**Published:** 2016-12-15

**Authors:** Haitao Zhu, Lirong Zhang, Yanfang Liu, Yuepeng Zhou, Kang Wang, Xiaodong Xie, Lian Song, Dongqing Wang, Chunlei Han, Qiuyun Chen

**Affiliations:** 1 Department of Radiology, Affiliated Hospital of JiangSu University, Zhengjiang, Jiangsu Province, 212001, China; 2 Department of Central Lab, Affiliated People’s Hospital of JiangSu University, Zhengjiang, Jiangsu Province, 212002, China; 3 Turku PET Centre, Turku University Hospital and University of Turku, Turku, Finland; 4 School of Chemistry and Chemical Engineering, Jiangsu University, Zhenjiang, Jiangsu Province, 212013, China

## Abstract

Hypoxia-induced cancer stem cells have been known to be involved in tumour metastasis, resistance to chemo/radio therapy and tumour recurrence. Magnetic Resonance Imaging is a widely used imaging tool for cancers in clinics and research. To develop T1-positive and T2-negative dual mode MRI agents for more comprehensive and accurate diagnostic information under hypoxic conditions, a hypoxia-inducible factor-1α based aptamer and Mn(II)-modified nanoparticles D-Fe_3_O_4_@PMn were synthesized and characterized. *In vitro and in vivo* studies show that D-Fe_3_O_4_@PMn NPs are biocompatible and less cytotoxic and can produce significant contrast enhancement in T1- and T2-weighted MR imaging. Furthermore, the D-Fe_3_O_4_@PMn NPs enable targeted dual-contrast T1- and T2-weighted MR imaging of cancer cells expressing high levels of HIF-1α and cancer stem cell-related proteins under hypoxic condition. In conclusion, NPs with HIF-1α and Mn(II) are promising diagnostic agents for dual-mode T1 and T2 imaging by targeting cancer stem cells as they are non-toxic and biocompatible.

Hypoxia is a common feature of most solid malignancies and is associated with the activation of angiogenesis, metastasis and recurrence potential^1^,^2^. Tumour hypoxia is an important negative prognostic factor. Hypoxic conditions eventually lead to the activation of hypoxia-inducible factor-1α (HIF-1α). HIF-1α plays a key role in many crucial aspects of cancer biology including angiogenesis, metabolic reprogramming, the epithelial-mesenchymal transition (EMT), invasion, metastasis, and resistance to radiation therapy and chemotherapy^3^,^4^. Recently, it has been confirmed that HIF-1α also plays a critical role in the specification and/or maintenance of cancer stem cells (CSCs)^5^,^6^. Cancer stem cells in tumour hypoxia regions are a response to tumour recurrence, local invasion, distant metastasis formation and treatment failure^7^,^8^. Based on this knowledge, a non-invasive imaging method is urgently needed to identify hypoxic microenvironments and measure the cancer stem cells within the tumour hypoxic region, which would help facilitate personalized medicine.

For tumour hypoxia imaging, molecular imaging will likely become an important *in vivo* imaging biomarker in the future by providing a snap shot of a primary tumour and metastatic disease and in subsequent treatment response^9^. Among the molecular imaging technologies, MRI is perhaps one of the most powerful imaging methods for its superiority in soft tissue contrast^10^. Moreover, MRI contrast agents can increase imaging sensitivity by enhancing the contrast in regions of interest (ROI) with brighter or darker signals in T1 or T2 images. Despite many attempts to modify MRI sequences (blood oxygen level-dependent, BOLD; proton MRI, ^1^H-MRI) or tailor contrast agents, there are still some challenges to overcome for more accurate measurements the hypoxic region in the tumour^11^,^12^,^13^. Shimpei reported that a Gd^3+^-based T1 contrast agent can be used as a hypoxia-sensitive probe *in vitro*^14^. However, this study was limited to the *in vitro* environment and no further study was reported. Additionally, many Gd^3+^ complexes have relatively short residence time in the vascular system and toxicity, especially causing nephrogenic systemic fibrosis^15^,^16^. Many attempts to overcome such obstacles in the use of modified T2-negative particles (e.g., Fe_3_O_4_, Fe_2_O_3_).These modification partly address the toxicity and rapid clearance from the organism. However, because of the negative contrast effect and magnetic susceptibility artefacts, the obtained dark areas in MR images are often confused with low signal arising from surrounding tissues^17^,^18^.

Because a single contrast agent has its own advantages and limitations, the combination of T1-positive and T2-negative agents into a single nanoprobe, creating T1/T2 dual-mode contrast agents (DMCAs) for MRI imaging, can give highly accurate information. The beneficial contrast effects are two-fold: the T1 imaging will give high tissue resolution while the T2 imaging gives high feasibility on the detection of diseases^19^. Di *et al*. reported that ultra-small super-paramagnetic iron oxide nanoparticles (USPIONs) with a core of less than 10 nm in diameter are capable of producing T1-positive and T2-negative images^20^. Compared to the regular single T1 or T2 contrast agents, the contrast effects of USPIONs are relatively weak at the same scanning condition, especially the T2 contrast effect. Zhou and co-workers reported that GdIO nanoparticles can act as a T1 and T2 mutually enhanced dual-modal contrast agent for MR imaging^21^. Shin *et al*. presented an artefact filtering dual-modal contrast agent that can eliminate the false errors in MRI imaging^22^. However, these nanoparticles lack a tumour targeting capability. Yang *et al*. designed the DMCAs modified with an RGD peptide for selectively targeting a_v_β_3_ over-expressed glioblastoma cancer cells *in vitro and in vivo*^23^. Recently, Kim and co-workers reported IO@MO as a T1/T2 dual-mode imaging probe that can be activated and can exhibit high sensitivity and effective silencing/activation of the MR contrast effect under a tumour-reducing environment^24^. The modified DMCAs can selectively accumulate and be activated under a tumour-specific microenvironment (the over-expressed receptors, acidic pH, hypoxia, etc.), which substantially increases the sensitivity and accuracy of tumour diagnosis. Based on this knowledge, we believe that the combined identification and mapping of tumour hypoxia with targeting the cancer stem cells subpopulation in hypoxic region would be realized using a modified dual-mode imaging probe. However, no such probe is currently available.

In this study, we developed a targeted T1/T2 dual-mode imaging probe that exhibits high sensitivity of the MR contrast effect for the special targeting of the cancer stem cell sub-population under hypoxic physiological conditions. PEG- and Mn(II)-modified magnetic nanoparticles (Fe_3_O_4_@PMn) were synthesized and assembled with oligonucleotide HIF-1α aptamers, forming D-Fe_3_O_4_@PMn NPs as an MRI diagnostic agent. An aptamer is a short oligonucleotide that can fold into a unique tertiary structure that recognizes a specific target ranging from small organic molecules to proteins to cells^25^,^26^. As a result of their unique advantages, such as low molecular weight, lack of immunogenicity, and higher specificity and stability, aptamers have become a notable class of targeting ligand for both diagnostics and therapeutics. Based on our previous studies, we used 5′-CTACGTGCT-3′ as the aptamer sequence for the specific recognition of HIF-1α^27^. An HIF-1α-based aptamer ensures the special accumulation of D-Fe_3_O_4_@PMn NPs in the hypoxic region. The physiochemical properties of those new materials are characterized using TEM, FT-IR, BET, and XRD. MTT assays were used to assess the cytocompatibility of the particles. The targeting specificity of D-Fe_3_O_4_@PMn NPs were evaluated by dual mode T1- and T2-weighted MR imaging *in vitro* and *in vivo* using human pancreatic carcinoma cell lines (Panc-1 and Bxpc-3) and a xenograft of Panc-1.

## Results

### Preparation and characterization of multifunctional nanoparticles

**Figure 1:**
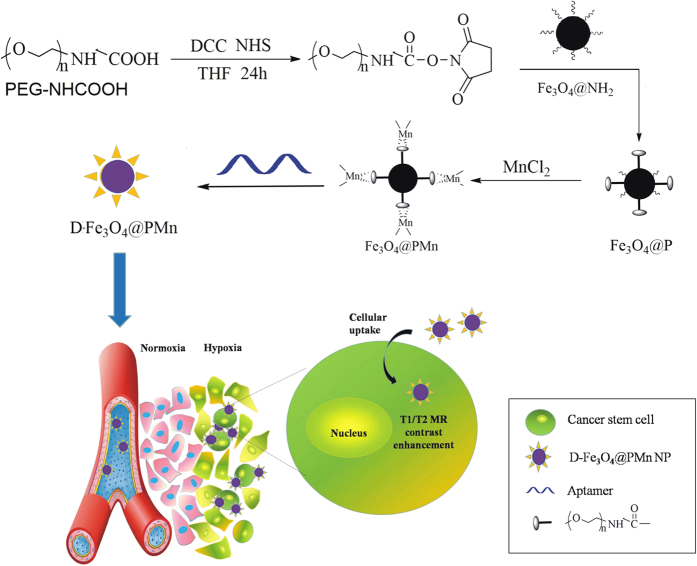
Schematic illustration of the protocol for establishment and function of D-Fe_3_O_4_@PMn NP.

**Figure 2:**
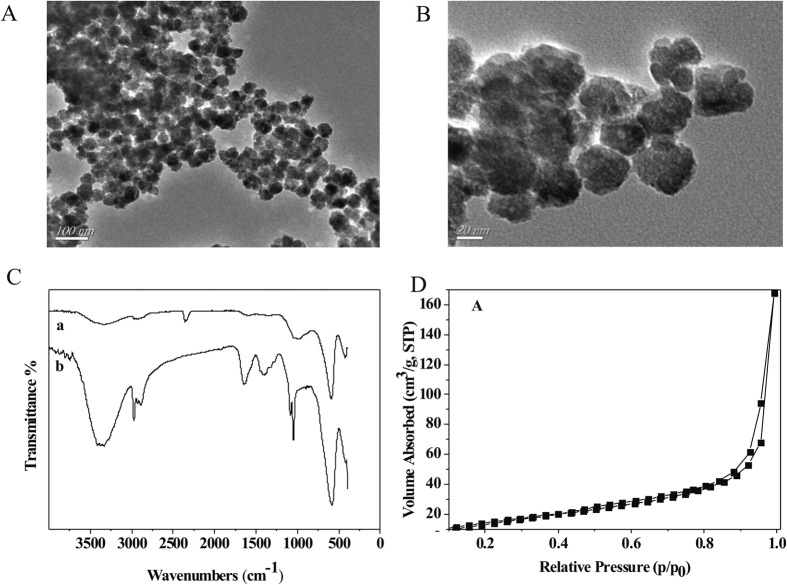
Characterization of Fe_3_O_4_ (a) and D-Fe_3_O_4_@PMn NPs. (**A,B**) TEM images of Fe_3_O_4_ (**A**) and D-Fe_3_O_4_@PMn NPs (**B**). (**C**) FT-IR spectra of Fe_3_O_4_ (a) and D-Fe_3_O_4_@PMn NPs (b), indicating surface functional groups. (**D**) Nitrogen adsorption–desorption data of D-Fe_3_O_4_@PMn, indicating the pore volume and pore diameter.

The synthesis route of D-Fe_3_O_4_@PMn is as shown in Fig. 1. Fe_3_O_4_ nanoparticles were first modified with PEGCOOH to improve biocompatibility, decrease non-specific affinity stability and allow for further coordination with Mn^2+^ to form Fe_3_O_4_@PMn nanoparticles. Then, negative HIF-1α aptamers bind on the surface of positive Fe_3_O_4_@PMn, which form D-Fe_3_O_4_@PMn nanoparticles. After magnetic separation, magnetic D-Fe_3_O_4_@PMn nanoparticles were obtained. The whole synthesis procedure was carried out under N_2,_ and the Fe_3_O_4_@PMn were obtained using a magnetic precipitation method, which avoids Fe_3_O_4_ being oxidized to Fe_2_O_3._ The D-Fe_3_O_4_@PMn was characterized by FT-IR, UV-Vis and TEM. Figure 2A shows a TEM image of the as-prepared Fe_3_O_4_ and D-Fe_3_O_4_@PMn. It is evident that the D-Fe_3_O_4_@PMn NPs were well dispersed without agglomeration. Based on the TEM observation, D-Fe_3_O_4_@PMn nanoparticles are round in shape with many holes on the surface, and their size is approximately 25–40 nm in diameter at room temperature. The D-Fe_3_O_4_@PMn spectrum showed -CH_2_- stretch signals around approximately 2897 cm^−1^, PEG-O stretch signals around 1049 cm^−1^, C = O stretch signals around 1638 cm^−1^, and N-H stretch signals around 3400 cm^−1^ (Fig. 2B). The nitrogen adsorption–desorption data of D-Fe_3_O_4_@PMn indicate that the pore volume and pore diameter are 0.2145 cm^3^/g and 5.507 nm, respectively. The BET surface area is 14.3463 m^2^/g, smaller than that of Fe_3_O_4_, which was 54.3864 m^2^/g (Fig. 2C and Table 1). The crystalline nature of the D-Fe_3_O_4_@PMn is verified by XRD analysis (Fig. S1). The saturation magnetization of Fe_3_O_4_@PMn is 65 emu/g. The pattern matches well with standard magnetite Fe_3_O_4_ reflection.

### Cytotoxicity of the nanoparticles

**Figure 3:**
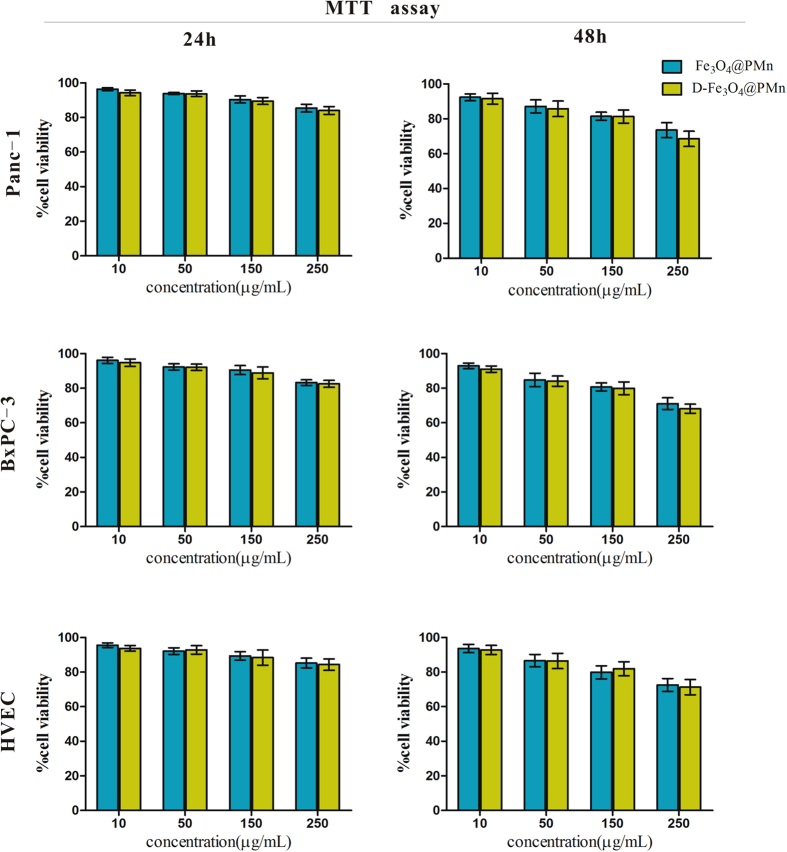
*In vitro* toxicity of non-target Fe_3_O_4_@PMn and D-Fe_3_O_4_@PMn NPs. Viability of Panc-1 cells in the presence of the samples with varied concentrations for 24 h (**A**) and 48 h (**B**). Viability of BxPC-3 cells in the presence of the samples with varied concentrations for 24 h (C) and 48 h (**D**). Viability of normal HVEC cells in the presence of the samples with varied concentrations for 24 h (**E**) and 48 h (**F**).

In this study, we chose pancreatic cancer cell lines (Panc-1 and BxPC-3) as the model. The cytotoxicity of nanoparticles is very important in their biomedical application both *in vitro and in vivo*. To examine the toxicity of D-Fe_3_O_4_@PMn, we incubated Panc-1 and BxPC-3 cells with different concentrations of non-target Fe_3_O_4_@PMn and D-Fe_3_O_4_@PMn for 24 h and 48 h. The cell viability was assessed using an MTT assay, and the results are shown in Fig. 3A–D. It is evident that the Panc-1 and BxPC-3 cells maintained greater than 80% cell viability after 24 h of incubation with 250 μg/mL non-targeted Fe_3_O_4_@PMn and D-Fe_3_O_4_@PMn. Although the cytotoxicity increased with the incubation time, over 60% cell viability was still obtained. Moreover, similar results can be observed when normal human vascular endothelial cells were co-cultured with nanoparticles (Fig. 3E,F). Compared to the non-target Fe_3_O_4_@PMn group, the cancer cell viabilities were lower when they were incubated with D-Fe_3_O_4_@PMn, which could be attributed to the excessive uptake of nanoparticles due to the aptamer targeting effect. However, with the cancer cell viabilities there were no significant differences between the Fe_3_O_4_@PMn group and the D-Fe_3_O_4_@PMn group. We therefore conclude that the new multifunctional nanoparticles are biocompatible.

### *In vitro* cellular uptake of the nanoparticles

**Figure 4:**
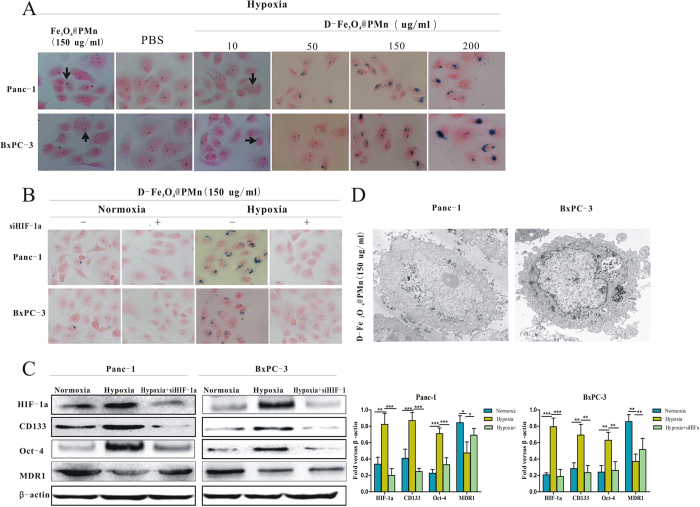
*In vitro* cellular uptake of the NPs. (**A**) Microphotographs of Prussian blue-stained Panc-1 and BxPC-3 cells in the presence of non-target Fe_3_O_4_@PMn and D-Fe_3_O_4_@PMn NPs with varied concentrations (0, 10, 50, 150, and 200 μg/mL). (**B**) Microphotographs of Prussian blue-stained Panc-1 and BxPC-3 cells co-cultured with D-Fe_3_O_4_@PMn NPs under normoxia and hypoxia with or without HIF-1α siRNA. (**C**) Western blot of the expression levels of HIF-1α, CD133, Oct-4 and MDR1 in the cells cultured under different conditions. (**D**) TEM images of the Panc-1 and BxPC-3 cells after an incubation with D-Fe_3_O_4_@PMn NPs (150 μg/mL).

The intracellular uptake of the multifunctional nanoparticles was demonstrated with Panc1 and BxPC-3 cultured under hypoxic condition with and without HIF-1α siRNA. The targeted uptake effect of nanoparticles was evaluated both by Prussian blue staining and TEM imaging. As shown in Fig. 4A, the labelled cells showed clusters of dense blue granules in the cytoplasm after 24 h of incubation, which indicates there is iron inside the cells. Compared to the PBS and non-target Fe_3_O_4_@PMn nanoparticles groups, the cells have taken up a larger number of D-Fe_3_O_4_@PMn. With an increase in the D-Fe_3_O_4_@PMn concentration, a higher uptake of nanoparticles was observed. Moreover, the peak uptake concentration was 150 μg/mL. However, when the cells were cultured under normoxic conditions or under hypoxic condition with HIF-1α siRNA, the results demonstrated the less efficient cellular uptake of D-Fe_3_O_4_@PMn (Fig. 4B). To discover the characteristics of the cells that have a high uptake of the nanoparticles, a western blot was carried out. As shown in Fig. 4C, the higher uptake subpopulation of cells expressed a high level of HIF-1α and two cancer stem cell-related proteins, CD133 and Oct-4, indicating a better targeting ability of the D-Fe_3_O_4_@PMn for pancreatic cancer stem cells. We also test the uptake of the D-Fe_3_O_4_@PMn by human vascular endothelial cells under normoxia condition. The results demonstrated that D-Fe_3_O_4_@PMn cannot be uptake by human vascular endothelial cells (Fig. S2). Since pancreatic cancer stem cells play a key role in predicting the biological aggressiveness of cancer, the target D-Fe_3_O4@PMn will provide additional benefits for a cancer patient’s therapy and diagnosis.

The intracellular distribution and uptake of D-Fe_3_O_4_@PMn were also characterized by TEM. As observed in Fig. 4D, majority of NPs were accumulated in vesicles and localized in the cytoplasm, which is in agreement with the Prussian blue staining. To further elucidating the mechanism of D-Fe_3_O_4_@PMn untaken by pancreatic cancer stem cells, we test the expression level of MDR1.The results demonstrated that pancreatic cancer stem cells in the hypoxia condition expressed lower level MDR1 than the other two groups(Fig. 4C).

### *In vitro* targeted dual mode MRI imaging

**Figure 5:**
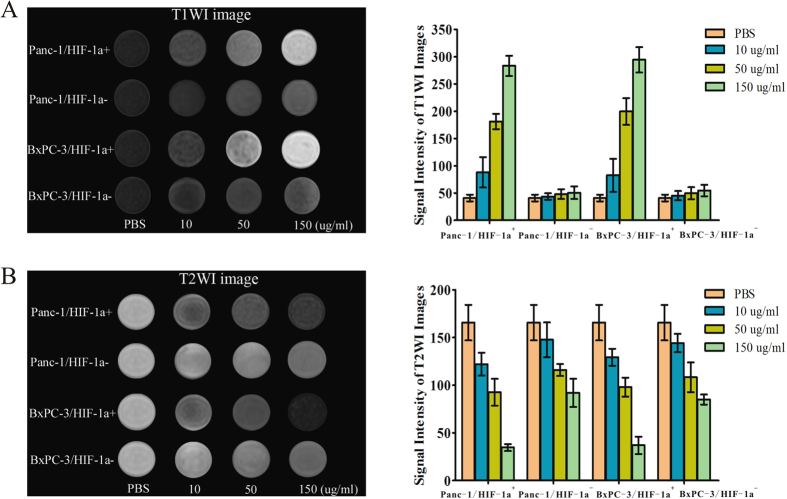
*In vitro* targeted MR imaging of cancer cells. (**A**) T1WI MR images and signal intensity analysis for T1WI MR images of Panc-1/HIF-1α^+^, Panc-1/HIF-1α^−^, BxPC-3/HIF-1α^+^ and BxPC-3/HIF-1α^−^ incubated with varied concentrations of D-Fe_3_O_4_@PMn NPs (0, 10, 50, and 150 μg/mL) for 24 h on 3.0 T MR system. (**B**) T2WI MR images and signal intensity analysis for T2WI MR images of Panc-1/HIF-1α^+^, Panc-1/HIF-1α^−^, BxPC-3/HIF-1α^+^ and BxPC-3/HIF-1α^−^ incubated with various concentrations of D-Fe_3_O_4_@PMn NPs (0, 10, 50, and 150 μg/mL) for 24 h on 3.0 T MR system. (HIF-1α^+^:cancer cells express high level of HIF-1α, HIF-1α^−^: cancer cells are transfected with HIF-1α siRNA and express a low level of HIF-1α).

**Figure 6:**
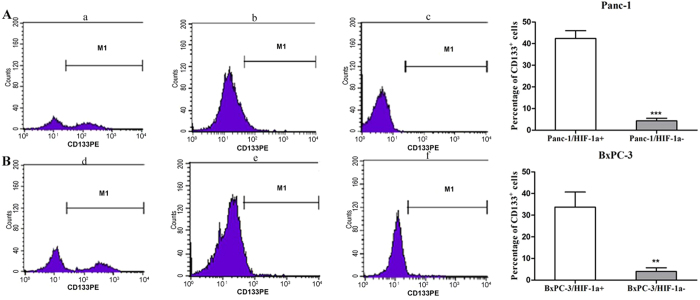
HIF-1α^+^ cancer cells are enriched in cancer stem cells. (**A**) The percentage incidence of the CD133^+^ subpopulation in Panc-1 cells lines under hypoxic conditions was assessed by flow cytometry. (**B**) The percentage incidence of the CD133^+^ subpopulation in BxPC-3 cells lines under hypoxic condition was assessed by flow cytometry. (a) Panc-1/HIF-1α^+^ group. (b) Panc-1/HIF-1α^−^ group. (c) IgG-PE antibody for parental Panc-1 cancer cells was used as a control. (d) BxPC-3/HIF-1α^+^ cancer cells group. (e) BxPC-3/HIF-1α^−^ group. (f) IgG-PE antibody for parental BxPC-3 cancer cells was used as a control. The experiment was repeated 3 times and the data were expressed as the mean ± standard deviation. The difference between these three groups was significant, ***P < 0.001.

Labelling cancer cells with nanoparticles enhances the cell-to-background contrast and makes them visible in MR images. To confirm the cancer cell special targeting ability and validate the dual mode MR imaging performance, the T1WI and T2WI MR imaging signal intensity of Panc-1 and BxPC-3 cells treated with and without D-Fe_3_O_4_@PMn were measured by a 3.0 T MRI system. Panc-1 cells with and without HIF-1α siRNA were incubated with different concentrations D-Fe_3_O_4_@PMn (10, 50, 150 μg/mL). First, we showed that the D-Fe_3_O_4_@PMn could be used for dual mode MR imaging. It is clear that the T1WI images got brighter with the increased concentration of D-Fe_3_O_4_@PMn. As a quantitative measurement of the signal intensity change displayed upon incubation with D-Fe_3_O_4_@PMn (150 μg/mL), the T1WI signal intensity of Panc-1/HIF-1α^+^ was 7 times higher than it was in the no-treatment control cells. Meanwhile, the T2WI images of the same cells darkened. The T2WI signal intensity of Panc-1/HIF-1α^+^ incubated with D-Fe_3_O_4_@PMn (150 μg/mL) was 5 times lower than the control cells without treatment (Fig. 5A and B). With increased concentrations of nanoparticles, we observed reduced signal in T2-weighted MR images and increased signal in T1-weighted MR images, indicating that these nanoparticles can act as both negative and positive contrast agents simultaneously. Second, we proved that the D-Fe_3_O_4_@PMn can target the HIF-1α-expressing cancer cells. HIF-1α siRNA-transfected Panc-1 cells (Panc-1/HIF-1α^−^) were treated with different concentrations of D-Fe_3_O_4_@PMn. As shown in Fig. 5A, the T1WI MR imaging signal intensity of Panc-1/HIF-1α^−^ cells incubated with D-Fe_3_O_4_@PMn (150 μg/mL) was only approximately 1.2 times higher than that of the control cells, and the T2WI MR imaging signal intensity was just 1 times lower than the control cells. Third, we further confirmed that the Panc-1/HIF-1α^+^ subpopulation is enriched in cancer stem cells. CD133 is one of the most important markers for CSCs in a variety of solid tumours, including pancreatic carcinoma. According to the previous reports, we designated CD133 positive cells as pancreatic cancer stem cells^28^,^29^. By FACS analysis, it was easily found that the proportion of CD133^+^ cells in the Panc-1/HIF-1α^+^ group (42.6 ± 3.10%) was much larger than in the Panc-1/HIF-1α^−^ group (4.39 ± 0.36%, a 10-fold decrease in CD133 positive population; Fig. 6). This result demonstrated that the Panc-1/HIF-1α^+^ subpopulation is enriched in CD133^+^ cancer stem cells. These inspiring observations indicated the great potential of D-Fe_3_O_4_@PMn as a target-specific and efficient dual-model contrast agent for MRI. Similar results can be observed from the BxPC-3/HIF-1α^+^ and BxPC-3/HIF-1α^−^ cells (Figs 5A,B and 6).

### *In vivo* targeted dual mode MR imaging

**Figure 7:**
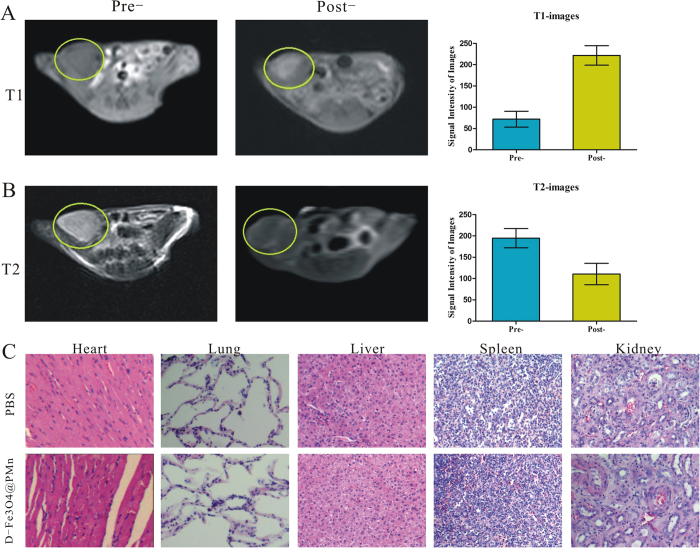
*In vivo* targeted dual mode MR imaging. (**A**) T1WI MR images and signal intensity analysis for T1WI MRI of Panc-1 tumour-bearing nude mice before and after injection of D-Fe_3_O_4_@PMn NPs in a 3.0 T MR system. (**B**) T2WI MR images and signal intensity analysis for T2WI MR images of Panc-1 tumour-bearing nude mice before and after injection of D-Fe_3_O_4_@PMn NPs in a 3.0 T MR system. (**C**) H&E staining images of major organs (heart, lung, liver, spleen, and kidney) of Panc-1 tumour-bearing nude mice in groups of PBS and D-Fe_3_O_4_@PMn NPs (the scale bar is 50 μm).

The targeted dual mode MR imaging of cancer cells using D-Fe_3_O_4_@PMn was further performed *in vivo* using Panc-1 subcutaneous tumour-bearing nude mice. Figure 7A shows the T1WI and T2WI images of tumour modes obtained pre- and 2 h post-injection, respectively. Compared with the image without NPs injection, the T1WI images of the tumour showed a significant bright enhancement effect 2 h following the NPs injection. A quantitative measure of the signal intensity change displayed the T1WI signal intensity in the tumour was approximately 3.5 times higher than before the injection. Meanwhile, T2WI images of the tumour section darkened after the injection. The T2WI signal intensity was approximately 2 times lower than before the injection.

To study any potential changes in organ morphology in the tumour-bearing mice, histopathological examinations of the major organs (heart, lung, liver, spleen, and kidney tissue) from each treatment group were carried out to determine the possibility of nanoparticle-induced toxicity. As shown in Fig. 7B, compared to the PBS group, the oedema and vacuolization in the heart was not found, the fibrosis and the inflammatory reaction in the lung and liver samples was not detected, and the glomerular and tubular structures in the kidney samples were also clearly displayed. All these results indicated that pathological changes were not found in D-Fe_3_O_4_@PMn group.

## Discussion

A solid tumour consists of highly proliferating tumour cells, which are characterized by hypoxic areas arising from an inequity between supply and consumption of oxygen. Hypoxia is a common phenomenon in malignant tumours. Many studies have demonstrated that hypoxia generally induces an aggressive tumour phenotype, such as increased invasiveness and resistance to chemotherapy. Pancreatic carcinoma is characterized by hypo-vascularization and extreme hypoxia in the early stage^30^. Hypoxia is very common in pancreatic carcinoma. Additionally, it was confirmed that several pancreatic cancer cell lines are enriched in cancer stem cells. In this study, we selected pancreatic cancer cell lines (Panc-1 and Bxpc-3) as our study model.

The tumour hypoxic environment may provide a site for the enrichment/expansion of the CSCs and successive rapid tumour progression. CSCs are undifferentiated cells with self-renewal ability that can differentiate into multiple lineages. CSCs are responsible for the genesis, growth, recurrence, and drug resistance of several tumours. This subpopulation of cells expresses distinct surface markers (CD133, CD44 and CD24) and proteins (Oct-4, Sox-2 and c-myc) and is thought to be involved in tumour origin, metastasis, recurrence and resistance to chemo-/radio-therapy^31^,^32^,^33^. CD133 is considered to be a CSC marker in various solid tumours including the pancreas. In this study, we designated CD133 positive cells as pancreatic cancer stem cells, while CD133 negative cells as non cancer stem cells. CSCs have been considered to be dependent on HIF-1a for survival and tumour growth under hypoxic condition^34^. Recently, several lines of evidence suggest that HIF-1a is involved in transcriptional regulation of CD133 through regulating CD133 promoter activity^35^,^36^. The CSC hypothesis has attracted much attention due to its potential in the development of CSC-related diagnostics and therapies. Nanotechnology-based approaches have demonstrated significant potential in drug delivery and cancer diagnosis, and many CSC-targeting nanomedicines are being introduced, developed and evaluated in various preclinical studies. However, most of these agents have characteristics that limit their clinical applications, such as an off-target effect, poor water solubility, short circulation time, inconsistent stability, and unsatisfactory bio-distribution. In this study, a HIF-1α-based multifunctional D-Fe_3_O_4_@PMn nanoparticle was synthesized. The cytotoxicity test revealed that the nanoparticles exhibited excellent biocompatibility *in vitro and in vivo*. Moreover, the new multifunctional NPs are stable under the studied pH values, temperature conditions and different aqueous media, which are essential for their further biomedical applications. As diagnostic agents, a major obstacle is their targeted delivery to specific disease sites in the body. One of the strategies to address this problem is the surface modification of the nanoparticles with target ligands. Aptamers are short oligonucleotides that can fold into unique tertiary structures that recognize a specific target ranging from small organic molecules to proteins and cells. As a result of their unique advantages, such as low molecular weight, lack of immunogenicity, and higher specificity and stability, aptamers have become a notable class of targeting ligand for both diagnostics and therapeutics. In the field of oncology, two aptamers, AS1411 and NOX-A12, inhibited tumour progression and reduced metastasis and drug resistance^37^,^38^. In this study, we used the HIF-1α-based aptamer as the guide to specially monitor the tumour hypoxia and target cancer stem cells. The tumour hypoxic condition tends to promote molecular changes and eventually lead to the activation of HIF-1α. HIF-1α expression is localized to the (hypoxic) pseudo-palisading cells that surround areas of necrosis. HIF-1α also plays a key role in the maintenance of cancer stem cells. Our previous studies demonstrated that the sequence 5′-CTACGTGCT-3′ was high-affinity for HIF-1α. In this study, we selected a new special sequence that can effectively bind to the surface of positively charged Fe_3_O_4_@PMn and display a high affinity for HIF-1α. Additionally, our results demonstrated that D-Fe_3_O_4_@PMn can be specially taken up by pancreatic cancer stem cells. To investigation mechanism for the special uptake of the NPs by the cancer stem cells, we test the expression level of MDR1 in the cancer cells. MDR1 is a member of the ABC subfamily. MDR1 is a classical pump, which binds substrates from the extracellular fluid and then transports these over the membrane. MDR1 also mediates the transport of various structurally unrelated compounds endogenous compounds^39^. We speculate that the non-target nanoparticles may be pumped out of the cell as a mechanism similar to the drug efflux mechanisms by MDR1. It is previously reported that cancer stem cells expressed higher level of MDR1. However, the cancer stem-like cells under hypoxic condition expressed lower level of MDR1.The exact mechanism needs further investigation. Previous studies have indicated that endocytosis plays a key role in nanoparticle uptake, particularly macropinocytosis and clathrin-mediated-endocytosis^40^,^41^,^42^. Macropinocytosis, the main form of endocytosis, is processed by forming intracellular vesicles by closing the waving cell surface ruffles back to the plasma membrane, which provides sufficient transportation for extracellular fluid bulks and macromolecules.

Molecular imaging has demonstrated its great value to the diagnosis of cancer at an earlier stage and revealed more information about the disease at the molecular and genetic level. Among the molecular imaging technologies, MRI is perhaps one of the most powerful methods due to its superiority in soft tissue contrast and capability to provide additional details regarding tissue function, structure, and blood perfusion. Because any single contrast agent has its own advantages and limitations, the combination of T1-positive and T2-negative agents would provide more comprehensive and accurate diagnostic information and has received great attention recently. *In vitro* and *in vivo* MR imaging studies also showed that the prepared multifunctional nanoparticles are an excellent targeted T1WI-positive and T2WI-negative MR contrast agent for cancer stem cells. In the *in vivo* study, we selected a 2 h scanning time point to obtain the maximum contrast effect. According to previous publications and our previous experiments, subcutaneous tumours have a poor blood supply. The D-Fe_3_O_4_@PMn nanoparticles may not enter the tumour site within a short period of time. Moreover, as the solid tumour has abnormal vasculature with leakiness combined with insufficient lymphatic drainage, the nanoparticles could accumulate in the tumour tissues via the enhanced permeability and retention effect (EPR).

## Conclusion

In the summary, we report a multifunctional D-Fe_3_O_4_@PMn nanoparticle. These high-sensitivity iron oxide nanoparticles can achieve simultaneous contrast enhancement in both T1-and T2-weighted magnetic resonance imaging and can target cancer stem cells located in hypoxic regions. This targeted new material can be potentially used as a molecular nanoprobe for tumour hypoxia diagnostic applications.

## Materials and Methods

### Materials and Instrumentation

Ferric chloride (FeCl_3_·6H_2_O), sodium acetate trihydrate (CH_3_COONa∙4H_2_O), ethylene glycol, ethylenediamine, sodium hydroxide (NaOH), tetrahydrofuran (THF), manganese (II) acetate tetrahydrate ((CH_3_COO)_2_Mn∙4H_2_O), dicyclohexylcarbodiimide (DCC), N-Hydroxysuccinimide and PEG-NHS (MW 2000) were purchased from Sinopharm Chemical Reagent Co. Ltd. (Shanghai, China). DNA was purchased from Sangon Biotech (Shanghai, China); the sequence is listed below. Prussian blue staining assay kit and MTT cell proliferation assay kit were purchased from Life Technologies (Shanghai, China). DMEM/F12, foetal bovine serum (FBS), trypsin-EDTA and penicillin/streptomycin were purchased from Invitrogen (CA, USA). Water was purified with a Millipore Milli-Q system (25 °C, 18.2 MΩcm, 7.2 × 10^−2^ Nm^−1^). All other reagents were purchased from Sigma-Aldrich (Shanghai, China) unless specifically mentioned otherwise.

The IR spectra were recorded on a Nicolet-470 spectrophotometer (Thermo Nicolet Corporation, USA) in the range of 4000–400 cm^−1^. The absorption spectra were recorded in the 200–800 nm regions using a Varian Cary 50-Bio UV-Vis spectrophotometer. The content of Fe and Mn in the nanoparticles was determined using an ICP-AES instrument (HORIBA Jobin Yvon, Longjumeau Cedex, France). TEM was carried out on JEOL JEM-200CX transmission electron microscope (Japan Electron Optics Laboratory Co. Ltd., Japan). X-ray diffraction (XRD) patterns of the NPs were obtained using a Philips double goniometer X’Pert system (PANalytical, St Laurent, QC) with Cu Kα radiation and recorded between 20° and 70° in the 2θ angle. Saturation magnetization was measured on an MPMS Xl superconducting quantum interferometer magnetometer (Quantum Design, American) at 77 K. *In vitro* and *in vivo* MR imaging were performed using 3.0 T magnet (Magnetom Trio Tim; Siemens, Erlangen, Germany).

### Synthesis of Fe_3_O_4_@PMn-aptamer nanoparticles (labelled as D-Fe_3_O_4_@PMn)

Fe_3_O_4_@NH_2_ nanoparticles were synthesized as previously reported^27^. Fe_3_O_4_@NH_2_ (300 mg) was mixed with PEG-NHS (2.0 g, 0.50 mmol) in DMF at 25 °C for 24 h, and then MnAc_2_4H_2_O (400 mg, 1.5 mmol) was added. The mixture was stirred for 8 h. After magnetic precipitation, Fe_3_O_4_@PMn was obtained.

To prepare the multifunctional Fe_3_O_4_@PMn-aptamer conjugate, HIF-1α aptamers (5′-ACAACA AGTATGTGGAGCAACTGTGTGG-3′) were used to react with Fe_3_O_4_@PMn obtained from the previous step. Briefly, aptamers were dissolved in Tris buffer (50 mM Tris, 100 mM NaCl). Six milligrams of Fe_3_O_4_@PMn NPs was dissolved in 300 μL phosphate-buffered saline (PBS, pH 7.4), to which 160 μL of the aptamer solution was added. The solution was subjected to gentle agitation for 2 h, followed by shaking for 16 h. The solution was centrifuged at 2,000 g for 10 min to separate the aptamer-NP conjugates from the unreacted aptamers. The precipitated D-Fe_3_O_4_@PMn NPs were resuspended in Tris buffer and finally filter-sterilized for subsequent cell culture experiments. The content of iron and manganese was 0.047 mg Fe/mg nanoparticle and 0.0725 mg Mn/mg nanoparticle, which were determined by the atomic absorption method.

### Cell culture and hypoxia environmental exposure

The human vascular endothelial cells (a generous gift from Dr. Yuming, Jiangsu University, Jiangsu, China) and pancreatic cancer cell lines (Panc-1 and BxPC-3, purchased from Cell Bank of China Academy of Sciences, Shanghai, China) were cultured in DMEM-F12 supplemented with 10% FBS, 100 U/mL penicillin and 100 U/mL streptomycin in a humidified atmosphere of 95% air with 5% CO_2_ at 37 °C. Cells were passaged with 0.25% trypsin/EDTA every 3 days.

For hypoxia induction, cancer cells were cultured in hypoxia chambers (Sanyo, containing 1% O_2_, 5% CO_2_, and 94% N_2_). Nitrogen gas was supplied to the chambers to induce a controlled reduced percentage of oxygen. To assess the specificity of the NPs, siRNA technology was used. When the cell density reached 50% confluence, the cultured cells were transfected with 40 nmol/L HIF-1α-specific siRNA (Suzhou Ribo Life Science CO., Ltd). Transfections were carried out according to the manufacturer’s instructions.

### Cytotoxicity experiment

To evaluate the cytotoxicity of the D-Fe_3_O_4_@PMn, the above cultured cells were seeded onto wells of a 96-well plate at a concentration of 1 × 10^4^ cells/well and incubated for 24 h. The non-target Fe_3_O_4_@PMn and D-Fe_3_O_4_@PMn were then added to the wells to achieve the pre-determined nanoparticle concentrations ranging from 0 to 250 μg/mL for 24 h and 48 h. The cells were then gently washed with fresh medium. Finally, 100 μL of a 1 mg/ml MTT solution was added to each well, and the plate was incubated for 2 h. Then, the DMSO solution was added to each well to dissolve the formed formazan crystals, and the absorbance at 490 nm was read using an ELISA reader (ELx800, BioTek Instruments, USA). Cell viability was calculated as the mean absorbance in treated cells divided by the mean absorbance of untreated controls (back ground absorbance is subtracted). Tests were performed in triplicate.

### Intracellular uptake experiments

To determine D-Fe_3_O_4_@PMn uptake by pancreatic cancer cells, both the Prussian blue staining technique and TEM imaging were used. For the Prussian blue staining technique, Panc-1 and BxPc-3 were cultured for 24 h in the presence of non-targeted Fe_3_O_4_@PMn (150 μg/mL, control) and D-Fe_3_O_4_@PMn (0, 10, 50, 150, 200 μg/mL) using 4-well chamber slides. Subsequently, the cells on the slide were washed twice with warm culture medium and fixed with 3.7% formaldehyde. To stain the cells on the slide, a mixture of 2.5% potassium ferrocyanide and 2.5% hydrochloric acid was used to incubate with the sample preparation for 20 min, and the slide was washed and counterstained with nuclear fast red. For TEM imaging, pancreatic cancer cells were treated with D-Fe_3_O_4_@PMn (150 μg/mL), followed by fixation in 2.5% glutaraldehyde at 4 °C for 1 h. The cells were then treated with 1% osmium tetroxide, dehydrated, and embedded in AGAR100 (Nanjing Medicine University, Nanjing) before they were sliced in ultrathin sections. The resulting preparation was observed using a JEOL JEM-200CX transmission electron microscope.

### Western blot analysis

Cells lysates were subjected to sodium dodecyl sulphate-polyacrylamide gel electrophoresis and transferred to polyvinylidene fluoride membranes (Merck Millipore, USA). Membranes were blocked with 5% (w/v) bovine serum albumin (BSA) in TBST for 1 h at room temperature and incubated overnight with primary antibodies at 4 °C. They were subsequently incubated with horseradish peroxidase-conjugated second antibodies. The immunoreactive bands were detected by chemiluminescence (ECL Plus, Merck Millipore) and relevant blots were quantified by densitometry using LANE-1D software. For immune detection, the primary antibody preparations were used as follows: rabbit-anti-human-Oct-4, rabbit-anti-human-CD133, rabbit-anti-human-HIF-1α and rabbit-anti-human-β-actin. All antibodies were obtained from Cell Signaling Technology, Inc. (Boston, USA). The secondary antibody preparations, either anti-rabbit or anti-mouse, were purchased from Boster biotechnology company (Wuhan, China).

### Flow cytometry analysis

Cells (5 × 10^6^) cultured under hypoxic and normoxic conditions were harvested, disaggregated into single-cell suspensions, and stained with 1.25 μg/ml mouse anti-human phycoerythrin (PE)-labelled CD133 (clone AC133, Miltenyi Biotec. Company, CA, USA). The antibody was incubated for 30 min at 4 °C in the dark. After incubation, the samples were washed with PBS and analysed by FACS Aria II (Becton Dickinson, USA). The sorting gate was established using cells stained with isotype control PE-conjugated mouse IgG1 antibody (Miltenyi Biotec. Company).

### *In vitro* targeted MR imaging of cancer cells

Panc-1 and BxPC-3 with and without HIF-1α siRNA were cultured under hypoxic conditions in 6-well plates for 24 h. When the cells reached 80% confluence, the medium was replaced with 3 mL fresh medium containing different concentrations of D-Fe_3_O_4_@PMn (10, 50, 150 μg/mL). After another 24 h, cancer cells were washed with PBS three times, digested and collected by centrifugation (1,000 rpm for 5 min). Finally, they were dispersed in 1 mL of 1% agarose in 1.5 mL Eppendorf tubes.

For MR imaging, the cell suspension in each tube was placed in a self-designed scanning holder and then scanned using a 3.0 T clinical MR instrument with a rodent receiver coil and turbo spin-echo (TSE) sequence. The imaging parameters for the T1-weighted (T1WI) gradient-echo were set as follows: TR = 600 ms, TE = 9.2 ms, FOV = 120 mm × 120 mm and slice thickness = 3.0 mm. The imaging parameters for the T2-weighted (T2WI) gradient-echo were set as follows: TR = 2000 ms, TE = 81.9 ms, matrix = 256 × 256, FOV = 100 mm × 100 mm and slice thickness = 3.0 mm. A region of interest (ROI) was selected to encompass cross sections of respective tubes. The mean T1 and T2 weighted signal intensity was measured for each cell sample.

### Animal studies

Nude mice were purchased from the Animal Center of Yangzhou University and maintained in Animal Center of Jiangsu University in compliance with the Guide for the Care and Use of Laboratory Animals (NIH Publication No. 85–23, revised 1996). The experimental protocols were approved by the Committee for Ethical Affairs of Jiangsu University (Zhenjiang, China), and the methods were carried out in accordance with the approved guidelines. To demonstrate the target imaging function of pre-prepared multifunctional D-Fe_3_O_4_@PMn, 20 Panc-1 subcutaneous tumour-bearing nude mice were enrolled in this study and divided into two equal groups randomly. For *in vivo* MRI measurements, tumour-bearing nude mice were administered D-Fe_3_O_4_@PMn (5 mg/mL) diluted in PBS at a dose of 20 mg/kg by intravenous injection through the tail vein. For *in vivo* imaging, the T1WI and T2WI MR images were obtained at pre- and 2 h post-injection of the nanoparticles. The 2D fast low angle shot imaging T1-weighted imaging (2D-FLASH T1WI) sequence parameters were set as follows: TR = 280 ms, TE = 3.02 ms, FOV = 100 mm×100 mm, slice thickness = 2.0 mm, matrix = 192 × 192, and flip angle = 90°. The turbo spin echo T2WI (TSE-T2WI) sequence parameters set as follows: repetition time = 3500 ms, echo time = 90 ms, flip angle = 150° and bandwidth = 260 Hz/pixel.

At day 10 after administration of the nanoparticles, the mice were sacrificed and the tissues, including the heart, liver, spleen and kidney, were removed, fixed in 10% formalin, paraffin-embedded and stained with haematoxylin and eosin (H&E) for histopathological evaluation.

### Statistical analysis

Data were expressed as the mean ± standard deviation (SD). Statistical differences were analysed by Student’s t test. A value of P < 0.05 was considered statistically significant, and the data are marked with (*) for P < 0.05, (**) for P < 0.01, and (***) for P < 0.001, respectively.

## Additional Information

**How to cite this article**: Zhu, H. *et al*. Aptamer-PEG-modified Fe_3_O_4_@Mn as a novel T1- and T2- dual-model MRI contrast agent targeting hypoxia induced cancer stem cells. *Sci. Rep.* **6**, 39245; doi: 10.1038/srep39245 (2016).

**Publisher’s note:** Springer Nature remains neutral with regard to jurisdictional claims in published maps and institutional affiliations.

## Supplementary Material

Supplementary Information

## Figures and Tables

**Table 1:** Nitrogen adsorption–desorption data of Fe_3_O_4_ and DFe_3_O_4_@P.

	Pore volume (cm^3^/g)	Pore diameter (nm)	BET surface area (m^2^/g)
Fe_3_O_4_	0.2562	2.254	54.3864
D-Fe_3_O_4_@PMn	0.2145	5.507	14.3463
