# Supplementary material for: Aptamer-PEG-modified Fe3O4@Mn as a novel T1- and T2- dual-model MRI contrast agent targeting hypoxia-induced cancer stem cells
Source: Sci Rep. 2016 Dec 15;6:39245. doi: 10.1038/srep39245 (PMC5157021; doi:10.1038/srep39245)
Supplement: Supplementary Information [file srep39245-s1.pdf]

Supplementary Information for:

Aptamer-PEG-coated Fe_3O_4@Mn as a novel T1- and T2- dual-model MRI contrast agent targeting hypoxia induced cancer stem cells

Haitao Zhu[1], Lirong Zhang[1], Yanfang Liu[4], Yuepeng Zhou [1], Kang Wang[1], Xiaodong Xie[1], Lian Song[1], Dongqing Wang[1*], Chunlei Han[1,5*], Qiuyun Chen[1,2*]

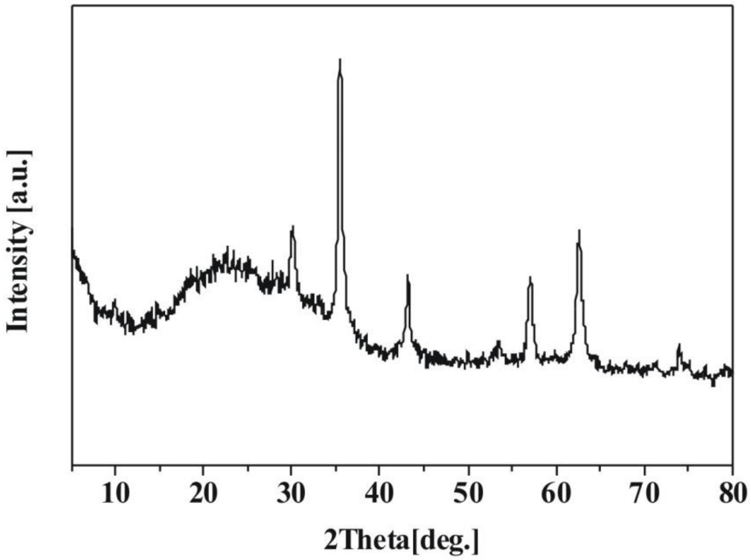

Fig.S1. X-ray diffraction pattern of D-Fe_3O_4@PMn.

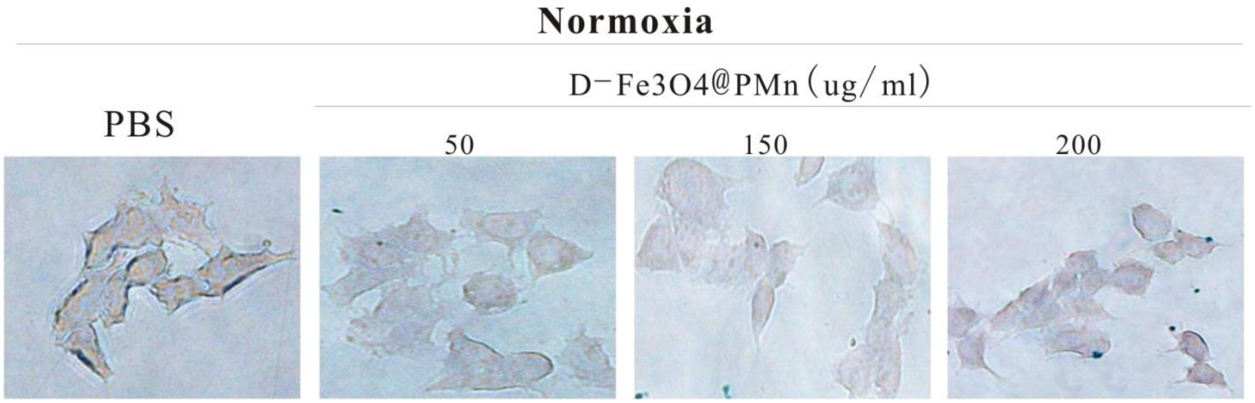

Fig.S2. Microphotographs of Prussian blue-stained human vascular endothelial cells in the presence of varied concentrations D-Fe_3O_4@PMn NPs.
